# Improved Protein Identification in Native Ambient Mass Spectrometry by the Integration of Proton Transfer Charge Reduction and Higher-Energy Collision Dissociation (PTCR-HCD MS^3^)

**DOI:** 10.1021/jasms.6c00165

**Published:** 2026-07-17

**Authors:** Rebecca L. Edwards, Oliver J. Hale, Sarah R. W. Vickers, Helen J. Cooper

**Affiliations:** School of Biosciences, 1724 University of Birmingham, Edgbaston, Birmingham B15 2TT, U.K.

## Abstract

Here, we demonstrate PTCR-HCD MS^3^ as a tool for improving protein identification in native ambient mass spectrometry (NAMS). NAMS involves the direct analysis of tissue sections with no or little sample preparation and no (solution-phase) separation postsampling. NAMS samples are therefore highly complex, comprising proteins, salts, and other biomolecules, and NAMS spectra are highly congested. PTCR MS has emerged as a useful strategy in top-down proteomics. Most commonly, it is applied as an adjunct MS^2^ step, providing molecular weight information to support sequence information provided by collision-based MS^2^ or it is applied as an MS^3^ step to simplify collision, photon, or electron-based MS^2^ by reducing *m*/*z* overlap of product ions. In this work, we show that the selection of charge-reduced PTCR product ions for subsequent HCD reduces the incidence of chimeric fragmentation spectra and improves confidence in protein identification in NAMS samples from mouse spinal cord, rat kidney, and cell-line-derived xenograft tissue. As well as removing interference from overlapping protein species, we show that PTCR-HCD MS^3^ also removes interference from overlapping lipid species which can similarly affect confident protein identification. The approach also allows identification of proteins that are masked at the MS1 level but are revealed following PTCR.

## Graphical abstract

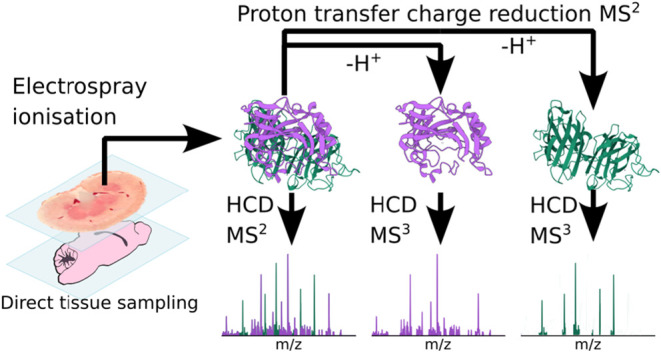

## Introduction

Native ambient mass spectrometry (NAMS) combines native mass spectrometry,^1–3^ top-down proteomics^4,5^ and ambient mass spectrometry^6,7^ for the direct detection and characterization of folded proteins and protein complexes from biological substrates in their native states.^8–11^ Liquid extraction surface analysis (LESA), nanospray desorption electrospray ionization (nano-DESI), and laser capture microdissection (LCM) coupled to high-resolution mass spectrometry have allowed for the characterization of intact proteins and protein assemblies, from small dimers to complexes over 231 kDa directly from tissue sections.^12–16^ Unlike methods that rely on denaturing or digestion for proteoform^17^ characterization, NAMS maintains noncovalent interactions, protein complex stoichiometry and post-translational modifications (PTMs) and provides simultaneous insight into spatial distribution and protein structure within biological substrates.^10,11,18,19^

The inherent complexity of biological tissues makes identification of proteoforms in NAMS challenging. NAMS spectra are information-rich, but extraction of this information is hampered by extensive peak overlap that results from the presence of large numbers of colocalized protonated proteoforms, salt-adducted (e.g., Na^+^, K^+^, NH_4_^+^) species and other co-ionized biomolecules (e.g., lipids). For unknown proteins and protein assemblies, intact mass is insufficient to identify and determine protein complex stoichiometry.^20^ With increasing sample complexity, overlap of charge state distributions increases, making spectral deconvolution and accurate intact mass calculation more challenging.^21^ Moreover, simultaneous fragmentation of multiple coisolated precursor ions produces heterogeneous fragment ion populations and chimeric MS^2^ spectra that can confound database search algorithms leading to low confidence or ambiguous identifications.^22^ These factors are particularly relevant when performing native ambient mass spectrometry directly from tissue, where lack of sample cleanup, sample complexity, and low analyte abundance further limit the ability to isolate and identify individual proteoforms.

To address these limitations, charge manipulation strategies have been introduced to reduce spectral overlap and improve proteoform identification.^23–26^ Among these strategies, proton transfer charge reduction (PTCR) MS has emerged as an effective method. PTCR MS involves gas-phase ion–ion reactions in which multiply protonated analyte ions undergo charge transfer with reagent anions.^27^ Product ions are shifted to lower charge states, resulting in the separation of overlapping multiply charged species and allowing resolution of protein mixture components. PTCR MS has been used in top-down proteomics to simplify spectra, improve intact mass calculation, enhance fragment ion interpretation and aid quantitation.^26,28–33^ Huguet et al. implemented a two-stage approach, with initial PTCR MS^2^ of ions isolated within a narrow *m*/*z* window to simplify proteoform mass calculation followed by higher-energy collision dissociation (HCD) or collisional induced dissociation (CID) MS^2^ on the same narrow *m*/*z* region to generate proteoform sequence information.^29^ In our laboratory, we routinely use PTCR MS^2^ in NAMS to determine intact mass of protein assemblies alongside HCD MS^2^ of the same precursors to obtain sequence fragments and thus protein identification^13,16,34^

While these studies demonstrate the power of PTCR and HCD combinations, they do not address the issue of chimeric MS^2^ spectra. Either PTCR MS^2^ and HCD MS^2^ are performed in parallel on the same precursor ions, or PTCR is performed as the MS^3^ step with the aim of simplifying MS^2^ results. Hunt and co-workers demonstrated that PTCR MS may be coupled to higher-energy collision dissociation (HCD) (i.e., PTCR-HCD MS^3^) in the analysis of a mixture of ribosomal proteins isolated from *Escherichia coli*.^35^ In this work, we demonstrate that PTCR-HCD MS^3^ reduces complexity and improves protein identification in NAMS. We show that this approach allows identification of otherwise unknown proteins, i.e., whose presence is not apparent from the MS^1^ spectra. At first consideration, these findings suggest that data-independent analysis (DIA) PTCR MS is a suitable approach for proteoform discovery; however, our results show that caution must be exercised as DIA PTCR-MS may overestimate the number of proteoforms in tissue samples and other samples of high complexity.

## Experimental Methods

### Materials

MS-grade water (catalog no. 10095164) was purchased from Fisher Scientific (Loughborough, UK). HPLC-grade ammonium acetate (catalog no. 15513351) was bought from J.T. Baker (Deventer, Netherlands). The detergents C_8_E_4_ (catalog number T3394) and C_10_E_5_ (catalog number 76436) were bought from Merck (Gillingham, UK). Mass spectrometer calibration was performed with FlexMix (catalog number A39239, Thermo Fisher Scientific, San Jose, CA) and ammonium hexafluorophosphate (AHFP, catalogue number 216593, Merck). Nitrogen (>99.995%) and helium (>99.996%) gases used on the mass spectrometers were obtained from BOC (Guildford, UK).

### Animal Tissues

Fresh frozen spinal cords from SOD1^G93A^ C57BL/6 transgenic mice^36^ were the kind gift of Dr. Richard Mead (University of Sheffield, UK). Tissue was dissected, snap frozen over dry ice on foil and stored at −80 °C. The lumbar enlargement was dissected from the frozen cord and bisected prior to sectioning.

Fresh-frozen cell-line derived xenograft tissue and rat kidney were the kind gift of Dr Richard Goodwin and Dr Andreas Dannhorn (AstraZeneca). *Xenograft tissue:* NCr Nude mice were implanted subcutaneously in the right flank with 5e6 NCI-N87 cells (in 100 μL of 1:1 ratio PBS/Matrigel). Tumours were grown for 9 days prior to treatment with trastuzumab emtansine and disitamab vedotin at 3 mg/kg before collection 48 h postdose. Tissue was snap frozen in isopentane over dry ice and stored at −80 °C. *Kidney tissue:* Kidney tissue from vehicle-dosed (0.5% hydroxypropyl methylcellulose (HPMC) and 0.1% tween 80 in water) adult male Han-Wistar rats (denoted BE002350–13/An2) was collected 6 h post dose, snap frozen in isopentane over dry ice and stored at −80 °C.

Tissues were sectioned with a CM1810 Cryostat (Leica Microsystems, Wetzlar, Germany) to 10 μm thickness at −23 °C before being thaw mounted onto glass slides. Sections were stored at −80 °C prior to analysis. Spinal cord and xenograft tissues were analyzed with no further preparation. Rat kidney sections were prepared for membrane protein analysis as described in.^37^ Briefly, kidney sections were thawed and washed three times with ∼200 μL of 200 mM ammonium acetate for 60 s. Sections were dried under vacuum between each wash cycle.

### Tissue Sampling

Tissue sections were sampled with manual liquid extraction surface analysis (LESA). Five μL of extraction solvent 200 mM ammonium acetate containing 0.5x CMC of detergent C_8_E_4_ (for spinal cord and xenograft tissue) or 2x CMC of detergent C_10_E_5_ (for kidney tissue) was aspirated into a gel loading tip. Approximately 0.5 μL was dispensed onto the tissue surface and the liquid junction maintained for 10–30 s before being reaspirated. Samples were transferred into pulled, gold-coated borosilicate nanoESI emitters (prepared with a micropipette puller (Sutter Instruments) and a sputter coater (Agar Scientific)).

### Mass Spectrometry

Mass spectrometry experiments were performed on either an Orbitrap Ascend Structural Biology Tribrid mass spectrometer (spinal cord and kidney tissue) or an Orbitrap Eclipse Tribrid mass spectrometer (xenograft tissue), (both Thermo Fisher Scientific, San Jose, CA).

The Orbitrap Ascend Structural Biology mass spectrometer was operated in ‘Intact Protein’ and ‘high pressure’ modes (F-IRM = 15 mTorr, B-IRM = 20 mTorr). The ion transfer tube temperature was set to 275 °C. For experiments on spinal cord, in-source dissociation was 80 V and the source CID compensation factor was 0.022. (Note: the source CID compensation factor acts to decelerate ions after they have been accelerated by in-source CID, which improves their transmission through the MP0 multipole on the Orbitrap Tribrid instruments.)^38^ For experiments on kidney tissue, in-source dissociation was 90 V and the source CID compensation factor was set to 0.03. For the full scan mode experiments, mass analysis was performed using the orbitrap analyzer at a resolution of 7500 at *m*/*z* 200 over an *m*/*z* range of 1000–8000. For MS^n^ analysis of CAH2 & SOD1 the electrospray voltage was 1000 V, AGC target was 2000% and maximum injection time was 3000 ms. For the identification of RACK1, the electrospray voltage was 1100 V, the AGC target was 10000% and the maximum injection time was 1500 ms.

The Orbitrap Eclipse mass spectrometer was operated in ‘Intact Protein’ mode at ‘high pressure’ (IRM = 20 mTorr). The ion transfer tube temperature was set to 275 °C. In-source collisional dissociation was set to 60 V and the source CID compensation factor was set to 0.04. For the SIM mode experiment, isolation was performed in the linear ion trap with a window *m*/*z* 3000 ± 1000 and mass analysis was performed in the orbitrap analyzer at a resolution of 7500 at *m*/*z* 200. For the identification of PPIA and RABP2, the electrospray voltage was 1200 V, the AGC target was 10000% and the maximum injection time was 1000 ms.

### Tandem Mass Spectrometry MS^n^

For tandem mass spectrometry experiments performed on the Orbitrap Ascend instrument, precursor ions were isolated by use of the quadrupole with isolation widths as specified in the main text for MS^2^ analyses followed by isolation of product ions in the linear ion trap for MS^3^ analyses. HCD collision voltage (MS^2^ and MS^3^ experiments) was between 90 and 120 V. The PTCR reagent was perfluoroperhydrophenanthrene and the reagent anion target was set to 2 × 10^5^ charges with a maximum fill time of 200 ms, typically resulting in a reagent injection time <1 ms. PTCR reaction times for individual experiments are given with relevant data. PTCR MS^2^ spectra were recorded at a resolution of 7500 @ *m*/*z* 200 in the orbitrap analyzer. HCD MS^2^ and PTCR-HCD MS^3^ spectra were recorded at a resolution of 240,000 @ *m*/*z* 200 in the orbitrap. Full Profile mode was on for experiments performed on spinal cord tissue.

For tandem mass spectrometry experiments on the Orbitrap Eclipse instrument, precursor ions were isolated in the linear ion trap with a width of 20 *m*/*z*. The PTCR reagent anion target was 2 × 10^5^ charges with a maximum fill time of 200 ms, typically resulting in a reagent injection time shorter than 1 ms. PTCR reaction time was 6.0 ms. PTCR MS^2^ data were recorded in the orbitrap analyzer at a resolution of 7500 @ *m*/*z* 200. For PTCR-HCD MS^3^ experiments, the HCD collision voltage was between 95–100 V and data were recorded in the orbitrap analyzer at a resolution of 240000 @ *m*/*z* 200.

### Data Analysis

PTCR MS^2^ spectra were deconvoluted by use of UniDec.^39^ Proteins were identified from HCD MS^2^ and PTCR-HCD MS^3^ data using ProSight PC or Prosight Native (both Thermo Fisher Scientific). Data were searched against the *Mus musculus, Rattus norvegicus* or *Homo sapiens* protein databases (downloaded from UniProt) as appropriate. Precursor tolerance was 1000 Da and fragment tolerance = 20 ppm (spinal cord; known proteins) or precursor tolerance 1500 Da and fragment tolerance = 15 ppm (xenograft, kidney; unknown proteins).

### Fragment Ion Simulation

Molecular formulas for product ions were generated from the protein sequence of interest by Protein Prospector [http://prospector.ucsf.edu/] and combined into .csv files using ProtPi [https://www.protpi.ch/]. Spectra were plotted using a Matplotlib-based Jupyter notebook.^40^

## Results

To demonstrate the utility of PTCR-HCD MS^3^, we considered the case of two endogenous protein complexes in mouse brain; the G93A mutant of human superoxide dismutase 1 (SOD1) and carbonic anhydrase 2 (CAH2). SOD1 exists as a dimer in which each subunit binds one Zn and one Cu ion (calculated holo-SOD1^G93A^ MW = 31980 Da). CAH2 is a monomeric protein which binds Zn (calculated holo-CAH2 MW = 29009 Da). Figure 1 shows the NAMS spectrum obtained following sampling of a section of spinal cord from a mouse model expressing the G93A mutant of human SOD1. The [M+10H]^10+^ ions of CAH2 (*m*/*z* 2901) are close in *m*/*z* to the [M+11H^+^]^11+^ ion of SOD1 (*m*/*z* 2907), Figure 1a. HCD MS^2^ of ions with *m*/*z* 2901.1 ± 2.5 reveals sequence fragments from both species along with subunits from dissociation of SOD1, Figure 1b. That is, even with the narrowest possible quadrupole isolation width available on the Orbitrap Ascend in high mass mode (5 *m*/*z*), the HCD MS^2^ spectrum is chimeric. A search of the data using ProsightPC identified CAH2 with 12% of fragments explained, Figure S1, Table S1, Supporting Information. Further interrogation of the data revealed the presence of a sodiated sequence ion of CAH2, ([b_40_+Na^+^+H^+^]^2+^). A chimeric MS^2^ spectrum comprising fragment ions from both SOD1 and CAH2, was also obtained following HCD MS^2^ of ions with *m*/*z* 2907.0 ± 2.5, Figure S2, Supporting Information. In that case, CAH2 was identified with 8% of fragments explained, see Figure S3, Table S2, Supporting Information. Sodiated fragment ions, [b_40_+Na^+^+H^+^]^2+^ and [y_81_+Na^+^+3H^+^]^4+^, were also observed in this spectrum. The PTCR MS^2^ spectrum of ions with *m*/*z* 2901.2 ± 2.5 revealed two series of charge-reduced product ions, Figure 1c, which were deconvoluted by use of UniDec to give a molecular weight of MW_meas_ = 29,003 Da for CAH2, in good agreement with the calculated MW (MW_calc_ = 29,009 Da), and MW_meas_ = 31925 Da for SOD1. The calculated MW for holo-SOD1^G93A^ is 31980 Da; however, demetalated SOD1, in which the dimer is bound to two metal ions (MW_calc_ = 31,850 Da) or three metal ions (MW_calc_ = 31,915 Da), is also known to exist.^19^ The measured MW for SOD1^G93A^ suggests that (potentially multiple) demetalated, salt-adducted species are being selected for fragmentation at this *m*/*z*.

**Figure 1:**
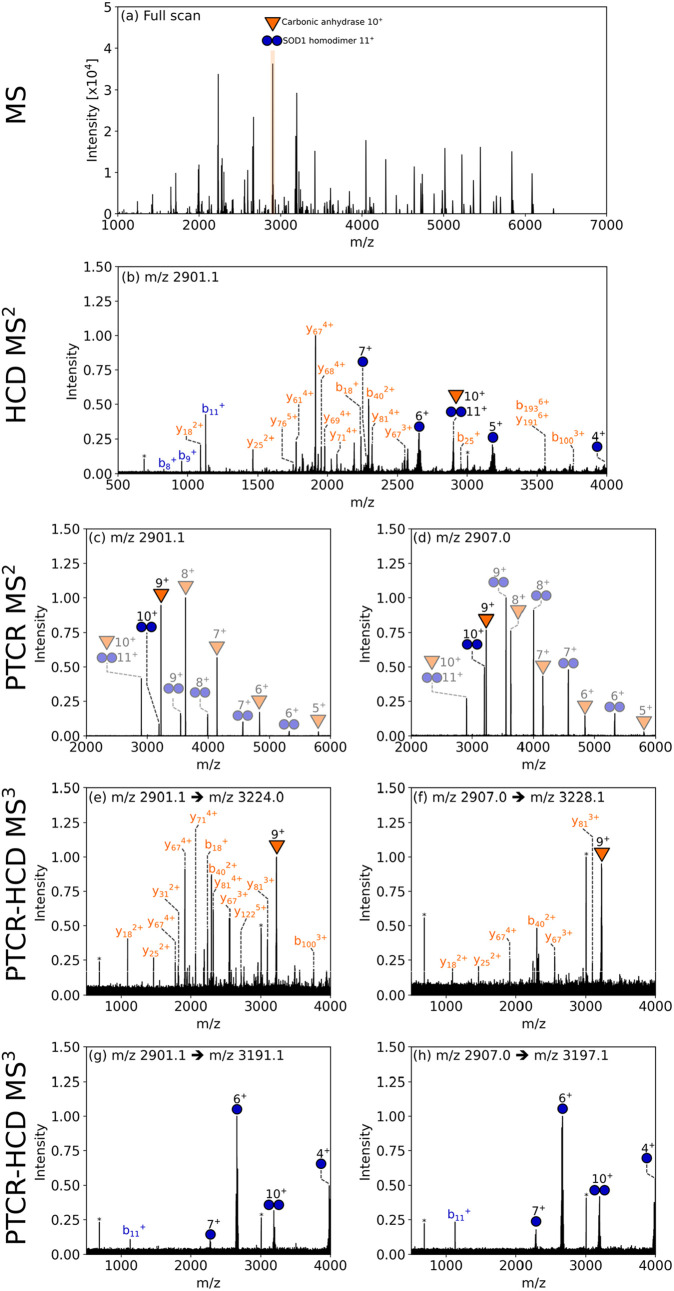
PTCR-HCD MS^3^ improves confidence in protein identification. (a) Full scan mass spectrum of LESA extract of SOD1^G93A^ mouse spinal cord; (b) HCD MS^2^ spectrum of ions with *m*/*z* 2901.1 ± 2.5, 90 V; (c) PTCR MS^2^ spectrum of ions with *m*/*z* 2901.1 ± 2.5, 4.5 ms; (d) PTCR MS^2^ spectrum of ions with *m*/*z* 2907 ± 2.5, 4.5 ms; (e) PTCR-HCD MS^3^ spectrum of ions with *m*/*z* 2901.1 ± 2.5, 4.5 ms → *m*/*z* 3224 ± 10, 115 V; (f) PTCR-HCD MS^3^ spectrum of ions with *m*/*z* 2907 ± 2.5, 4.5 ms → *m*/*z* 3228.1 ± 10, 115 V, (g) PTCR-HCD MS^3^ spectrum of ions with *m*/*z* 2901.1 ± 2.5, 4.5 ms → *m*/*z* 3191.1 ± 10, 90 V, (h) PTCR-HCD MS^3^ spectrum of ions with *m*/*z* 2907 ± 2.5, 4.5 ms → *m*/*z* 3197.1 ± 10, 90 V. Each HCD MS^2^ and PTCR-HCD MS^3^ spectrum comprises the average of 100 transients. Ion–ion reaction time noted in “ms” for each experiment.

Provided that the precursor ions exist in different charge states, the benefit of PTCR MS^2^ is that overlapping species become separated through generation of their charge-reduced product ions. These product ions can then be selected for further fragmentation. The fragmentation spectrum obtained following selection of the 9+ PTCR product ion of CAH2 (*m*/*z* 3224) and subsequent HCD (PTCR-HCD MS^3^) is shown in Figure 1e. A search of these data using ProSight PC identified CAH2 with 20% of fragments explained, Figure S4, Table S3, Supporting Information. No salt-adducted CAH2 fragments were observed, and importantly no confounding fragments from SOD1 were observed. Similarly, HCD fragmentation of the 10+ PTCR product ion of the SOD1 species (*m*/*z* 3191) results in detection of the SOD1 monomer subunits in various charge states, and b_11_^+^ sequence ions deriving from a site of known preferential cleavage, without interference from CAH2 fragments, Figure 1g.

The PTCR MS^2^ spectrum of ions with *m*/*z* 2907 ± 2.5 (Figure 1d) also revealed the presence of two series of charge-reduced product ions. Deconvolution by use of UniDec gave molecular weights of MW_meas_ = 29044 Da and MW_meas_ = 31988 Da. The latter is in good agreement with the calculated MW for holo-SOD1^G93A^ (M*W*_calc_ = 31980 Da); however, there is a mass difference of ∼+43 Da between the calculated and measured MW of the putative CAH2. The 9+ PTCR product ions of the putative CAH2 (*m*/*z* 3228) were selected for HCD, Figure 1f, and the resulting fragments were searched using ProSight PC. CAH2 was identified with 12% of fragments explained, and manual analysis revealed the presence of the sodium-adducted [b_40_+Na^+^+H^+^]^2+^ fragment ion, see Figure S5, Table S4, Supporting Information. No fragments of SOD1 were observed in this mass spectrum. HCD of the 10+ PTCR product ions of SOD1^G93A^ (PTCR-HCD MS^3^) results in generation of the SOD1 monomer subunits together with the b_11_^+^ sequence fragment, Figure 1h.

The results above illustrate the advantages of combining PTCR and HCD to improve confidence in protein identifications. For both initial precursors (*m*/*z* 2901 and 2907), CAH2 was identified with higher % explained fragments from the PTCR-HCD MS^3^ analysis than the HCD MS^2^ analysis. Essentially, PTCR provides a cleanup step and eliminates or reduces chimeric fragmentation spectra in the HCD step. The same was true for SOD1^G93A^ in these analyses. It should be noted that HCD of the SOD1 dimer is known to result in the dissociation to the monomer subunits and a prominent sequence fragment (b_11_^+^), and that searching these data against protein databases does not result in definitive identification.^41^ Nevertheless, the presence of these fragments provides a confident manual assignment.

These results also highlight the challenges associated with salt-adducted species for proteoform discovery in NAMS analyses. Precursor *m*/*z* 2901 corresponds to the 10+ charge state of protonated CAH2. Precursor *m*/*z* 2907 corresponds to the 11+ charge state of the protonated SOD1^G93A^ dimer, but quadrupole isolation of those species also selected some of the salt-adducted tail of the 10+ CAH2 peak. The difference between the mass calculated for protonated CAH2 and the measured mass (+∼43 Da) suggests that the species has, for example, two sodium adducts bound (although other adduct combinations are possible). Figure 2a shows a comparison of the PTCR-HCD MS^3^ spectra obtained from the two precursors (*m*/*z* 2901 and 2907). Sodium-adducted fragments are observed for precursor *m*/*z* 2907 but not for precursor *m*/*z* 2901. Simulated fragment spectra are shown in red for comparison. If one simply considers the deconvoluted molecular weights, PTCR MS^2^ of *m*/*z* 2901 and *m*/*z* 2907 appears to show the presence of two separate proteoforms with MW_meas_ 29003 and 29044 Da; however, our results show that these are differently adducted versions of the same proteoform. This point is important to note when using approaches such as data independent analysis (DIA) PTCR. DIA PTCR involves automated PTCR analysis of successive *m*/*z* windows across an *m*/*z* range to facilitate proteoform discovery. The approach has been successful for interrogating the molecular weight heterogeneity in glycosylated biotherapeutic preparations^42^ and offers an attractive proposition for deepening proteome coverage in NAMS. Nevertheless, care must be taken not to overestimate the number of proteoforms detected. To illustrate, Figure 2b shows an example peak in a NAMS spectrum. The peak is broad, the result of salt adduction and *potentially* the presence of a number of proteoforms. (For example, the 29+ charge state of the 231 kDa pyruvate kinase tetramer, sampled from brain tissue, recorded at resolution 7500 at *m*/*z* 200, is typically ∼ 20 *m*/*z* wide at its base).^16^ Overlaid are three possible isolation windows, w1, w2 and w3. Application of these three isolation windows would result in three apparent precursor ion peaks with *m*/*z* offset by the width of the isolation windows. Deconvolution of the resulting PTCR MS^2^ spectra for these three “precursors” would suggest the presence of three proteoforms, all with different MWs (MW1, MW2 and MW3). To avoid overestimation in this manner, the identity of the PTCR product ions must be confirmed by MS^3^.

**Figure 2:**
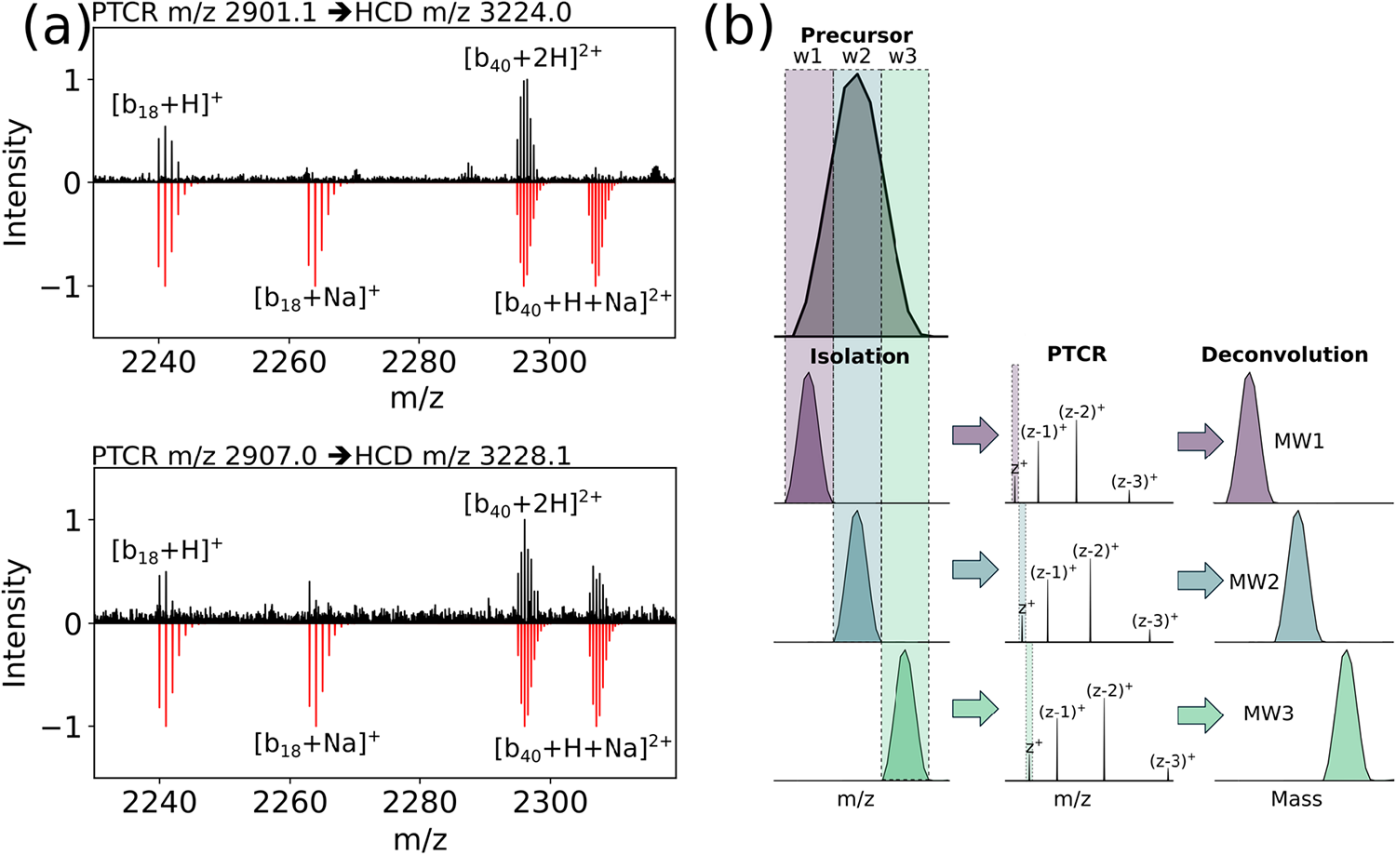
(a) Comparison of region of PTCR-HCD MS^3^ spectra from precursor ions with *m*/*z* 2901.1 (top) and *m*/*z* 2907 (bottom). Simulated fragment ion spectrum is shown in red. (b) Representative illustration of the challenges of DIA-PTCR for proteoform discovery. The precursor peak is wider than the quadrupole isolation window, which results in segments of the peak being subjected to PTCR. Deconvolution of the PTCR MS^2^ spectra produces three molecular weights (MW1, MW2 and MW3) that could be erroneously interpreted as different proteoforms.

The findings above show the advantages of combining PTCR and HCD to improve confidence in protein identification using two well-established examples of proteins detected in NAMS of mouse spinal cord tissue. The approach may also be applied for identification of unknown proteins and proteoforms. Figure 3a shows a NAMS spectrum obtained from cell-line derived xenograft tissue. The previously unidentified peak at *m*/*z* 2594 ± 10 was selected for PTCR MS^2^, which revealed the presence of two overlapping precursor ions with charge states 6+ and 7+, Figure 3b. Deconvolution of the charge-reduced product ions confirmed that the two precursor ions had molecular weights 15562 Da (6+ precursor) and 18186 Da (7+ precursor). The 6+ PTCR product ion of the species with MW = 18 kDa (*m*/*z* 3032) was selected for HCD (Figure 3c) and the product ions were searched against the human protein database. The protein peptidyl-prolyl cis–trans isomerase A (PPIA) was identified, Figure S6, Table S5, Supporting Information, with a mass shift, Δ = 305 Da, from the calculated MW (MW_calc_ = 17881) consistent with glutathionylation at Cys51, Cys61 or Cys114. Confidence in the protein assignment is high despite the low sequence coverage as the fragments observed derive from known preferred cleavage sites (C-terminal to Asp/Glu).^43,44^ PPIA has previously been identified in rat kidney in both the unmodified and N-acetylated form,^10,14^ but this is the first identification of the human glutathionylated proteoform by NAMS.

**Figure 3:**
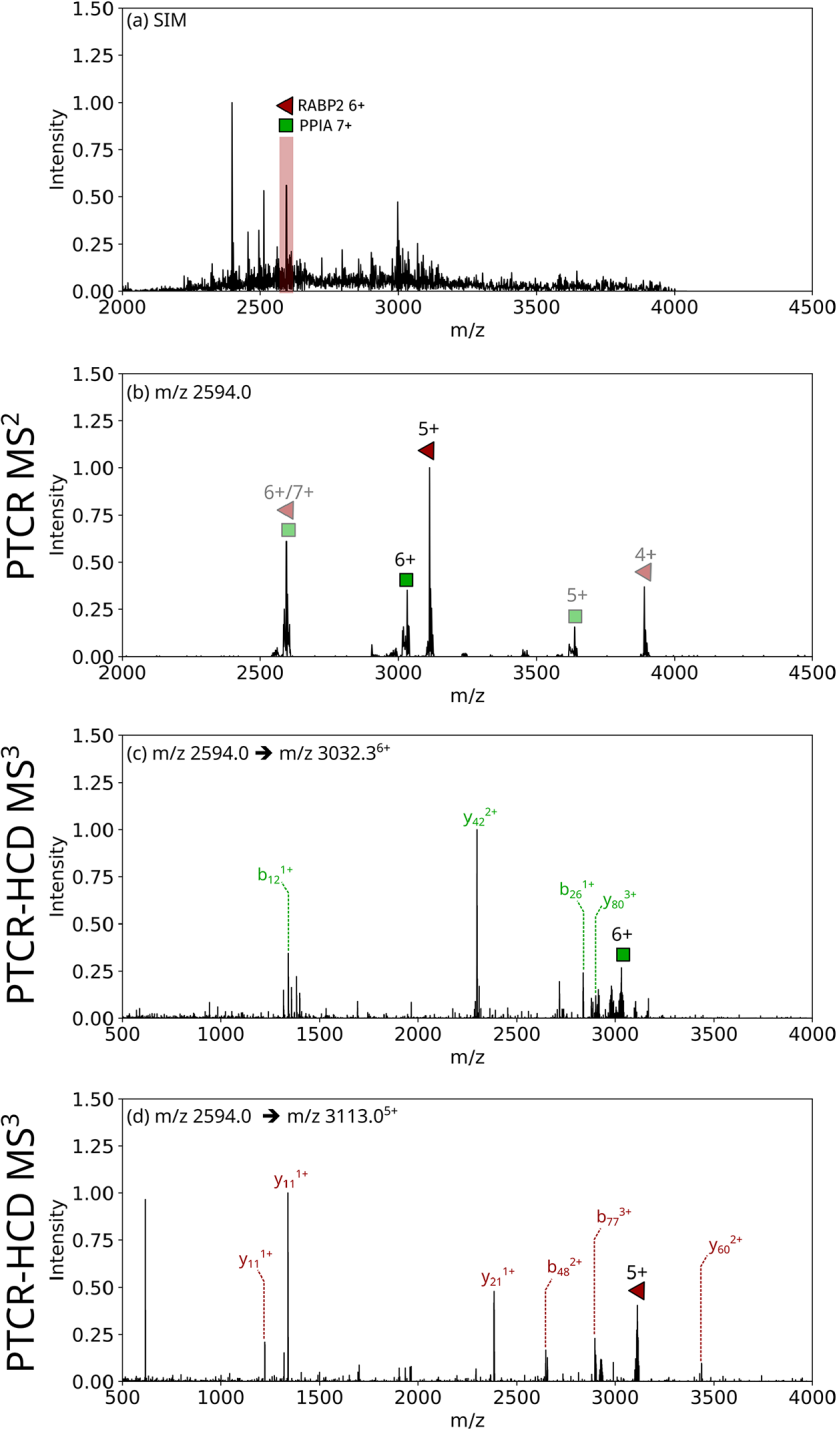
PTCR-HCD MS^3^ enables identification of previously unidentified proteins. (a) Wide SIM-mode (*m*/*z* 3000 ± 1000) mass spectrum of LESA extract of xenograft tissue; (b) PTCR MS^2^ spectrum of ions with *m*/*z* 2594.0 ± 10, 6.0 ms reveals the presence of two overlapping species with MWs 15.5 kDa (red triangles) and 18.1 kDa (green squares). (c) PTCR-HCD MS^3^ spectrum of ions with *m*/*z* 2594.0 ± 10, 6.0 ms → *m*/*z* 3032.3 ± 10, 95 V, select fragments labeled. (d) PTCR-HCD MS^3^ spectrum of ions with *m*/*z* 2594.0 ± 10, 6.0 ms → 3113.0 ± 10, 100 V, select fragments labeled. Each PTCR-HCD MS^3^ spectrum comprises the average of 1000 transients averaged over 13 min. Ion–ion reaction time noted in “ms” for each experiment.

Similarly, the 5+ PTCR product ions of the 15.5 kDa species (*m*/*z* 3113) were selected for HCD, Figure 3d. The MS^3^ fragment ions were searched against the human database and the protein cellular retinoic acid-binding protein 2 (RABP2) was identified, Figure S7, Table S6, Supporting Information. RABP2 has not previously been identified by NAMS in any form from any species.

A fundamental feature of NAMS is the lack of sample cleanup following tissue sampling. This aspect is important as it ensures there is no disruption of protein structure and therefore that the NAMS spectrum is a true (or close-to-true) reflection of the native environment. In consequence, the baselines of NAMS spectra include contributions from other biomolecules present in the tissue, such as lipids, in addition to multiple and numerous overlapping protein species. Even in the absence of overlapping protein species, PTCR-HCD MS^3^ offers advantages for separation of these other biomolecular species. For example, Figure 4a shows a NAMS spectrum obtained from rat kidney washed using ammonium acetate and sampled with a solvent containing 2x critical micelle concentration of detergent with the aim of identifying membrane and membrane-associated proteins. The previously unidentified peak at *m*/*z* 3181 ± 5 was selected for HCD MS^2^, Figure 4b, and the protein was identified as small ribosomal subunit protein 1, RACK1 (15% fragments explained), Figure S8, Table S7, Supporting Information. The HCD MS^2^ spectrum contains abundant peaks in the range *m*/*z* 700–850, corresponding to phospholipids, and *m*/*z* 1400–1700 (nonspecific phospholipid dimers). For an unidentified protein, a possible interpretation is that these are singly charged sequence fragments and their presence can lead to incorrect protein identification. Alternatively, they could be interpreted to have been noncovalently associated with the protein in the cell membrane environment. In fact, deconvolution of the PTCR MS^2^ spectrum of ions with *m*/*z* 3181, Figure 4c, confirms the measured MW is 34990 Da, in good agreement with the calculated MW (34988 Da). It is therefore more likely that the additional peaks in the HCD MS^2^ spectrum arise from lipid clusters that were isolated in the *m*/*z* window along with the protein of interest. For example, at *m*/*z* 3181, it is conceivable that clusters composed of phosphatidylcholines (2x 781.6 and 2x 809.6 Da ≈ *m*/*z* 3183^+^) are present but many plausible combinations exist. The PTCR-HCD MS^3^ spectrum of the 10+ PTCR product ion (*m*/*z* 3499) reveals no peaks corresponding to lipids (Figure 4d). A search of these data confirms the identity of the protein as RACK1 (32% fragments explained), Figure S9, Table S8, Supporting Information. That is, the confidence in the protein assignment was improved through use of PTCR-HCD MS^3^ as the PTCR step removed the confounding lipids from the HCD mass spectrum and avoids ambiguity over protein–lipid interactions.

**Figure 4:**
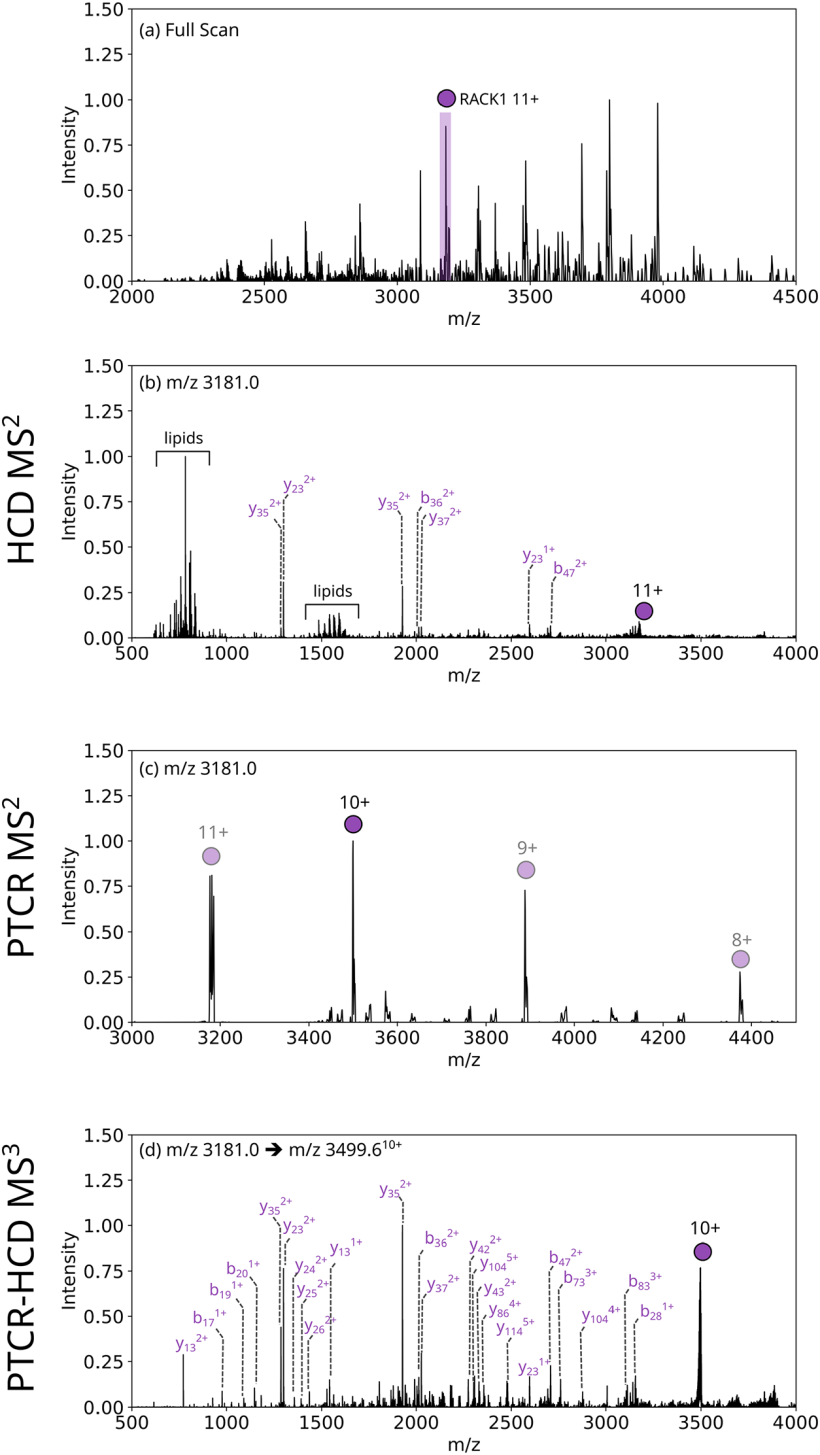
(a) Full scan mass spectrum obtained following LESA sampling of washed rat kidney tissue; (b) HCD MS^2^ spectrum of ions with *m*/*z* 3181.0 ± 5, 105 V. Peaks corresponding to lipids are observed; (c) PTCR MS^2^ spectrum of ions with *m*/*z* 3181.0 ± 10, 3 ms; (d) PTCR-HCD MS^3^ spectrum of ions with *m*/*z* 3181.0 ± 5, 3 ms → 3499.6 ± 10, 120 V. Each HCD MS^2^ and PTCR-HCD MS^3^ spectrum comprises the average of 100 transients. Ion–ion reaction time noted in “ms” for each experiment.

## Conclusion

Our findings demonstrate that PTCR-HCD MS^3^ is a useful tool for protein identification in native ambient mass spectrometry. The intrinsic complexity of the sample can be addressed through gas-phase fractionation of the sample by PTCR prior to HCD. The approach eliminates, or reduces, the occurrence of chimeric spectra which comprise sequence fragments from multiple protein precursor ions, or lipid clusters coisolated with the protein precursor ions. As a result, improved confidence in protein identifications is observed.

Nevertheless, it is worth noting that PTCR-HCD MS^3^ is inefficient in its use of PTCR MS^2^ product ions for MS^3^ as only a single charge state is selected. Limited control of the ion–ion reactions results in a product ion charge state distribution, essentially diluting the MS^3^ precursor ion population. Techniques such as ion parking would address this shortcoming by enabling PTCR product ions to be concentrated into a single charge state prior to MS^3^, providing a greater number of ions for fragmentation and more abundant MS^3^ product ions.^35^ Alternatively, multiplexed precursor ion selection in the linear ion trap could be used to isolate multiple charge states from the MS^2^ product ion series in parallel for a similar effect.^45^ Such approaches will be investigated in the future.

The PTCR step can also reveal the presence of otherwise unsuspected protein precursors which can then be identified, as in the example of RABP2 which was masked by PPIA in the MS^1^ spectrum. Our results also highlight that, while a promising approach for proteoform discovery in complex samples, automated PTCR analyses may result in misinterpretation of salt-adducted species and subsequent overestimation of the number of proteoforms present.

## Supplementary Material



## Notes

Sequence fragment maps and fragment ions assignments from MS^n^ spectra; HCD MS^2^ spectrum (PDF)
