# Supplementary material for: Improved Protein Identification in Native Ambient Mass Spectrometry by the Integration of Proton Transfer Charge Reduction and Higher-Energy Collision Dissociation (PTCR-HCD MS3)
Source: J Am Soc Mass Spectrom. 2026 Jul 17;37(8):1971–80. doi: 10.1021/jasms.6c00165 (PMC13450391; doi:10.1021/jasms.6c00165)
Supplement: Supplementary file 1 [file js6c00165_si_001.pdf]

Supporting Information

for

Improved protein identification in native ambient mass spectrometry by integration of proton transfer charge reduction and higher-energy collision dissociation (PTCR-HCD MS^3)

Rebecca L. Edwards, Oliver J. Hale, Sarah R.W. Vickers and Helen J. Cooper*

School of Biosciences, University of Birmingham, Edgbaston, Birmingham B15 2TT, UK.

*To whom correspondence should be addressed: h.j.cooper@bham.ac.uk

Table of contents

Figure S1. Sequence fragment map for CAH2 obtained following HCD MS^2 of m/z 2901.1 ±2.5. 3

Table S1. Fragment ions observed from CAH2 following HCD MS^2 of m/z 2901.1 ±2.5. 4

Figure S2. HCD MS^2 spectrum of m/z 2907 ±2.5 ions obtained from mouse spinal cord tissue section. 5

Figure S3. Sequence fragment map for CAH2 obtained following HCD MS^2 of m/z 2907 ±2.5. 5

Table S2. Fragment ions observed from CAH2 following HCD MS^2 of m/z 2907 ±2.5. 6

Figure S4. Sequence fragment map for CAH2 obtained following PTCR-HCD MS^3 of m/z 2901.1 ±2.5 → m/z 3224. 6

Table S3. Fragment ions observed from CAH2 following PTCR-HCD MS^3 of m/z 2901.1 ±2.5 → m/z 3224. 7

Figure S5. Sequence fragment map for CAH2 obtained following PTCR-HCD MS^3 of m/z 2907 ±2.5 → m/z 3228. 8

Table S4. Fragment ions observed from CAH2 following PTCR-HCD MS^3 of m/z 2907 ±2.5 → m/z 3228. 8

Figure S6. Sequence fragment map for glutathionylated PPIA (P62937) obtained following PTCR-HCD MS^3 of m/z 2594 ±10 → m/z 3032. 9

Table S5. Fragment ions observed from glutathionylated PPIA (P62937) obtained following PTCR-HCD MS^3 of m/z 2594 ±10 → m/z 3032. 9

Figure S7. Sequence fragment map for RABP2 (P29373) obtained following PTCR-HCD MS^3 of m/z 2594 ±10 → m/z 3113. 9

Table S6. Fragment ions observed from RABP2 (P29373) obtained following PTCR-HCD MS^3 of m/z 2594 ±10 → m/z 3113. 10

Figure S8. Sequence fragment map for RACK1 (P63245) obtained following HCD MS^2 of m/z 3181 ±5. 10

Table S7. Fragment ions observed from RACK1 (P63245) following HCD MS^2 of m/z 3181 ±5. 11

Figure S9. Sequence fragment map for RACK1 (P63245) obtained following PTCR-HCD MS^3 of m/z 3181 ±10 → m/z 3499. 12

Table S8. Fragment ions observed from RACK1 (P63245) following PTCR-HCD MS^3 of m/z 3181 ±10 → m/z 3499. 13

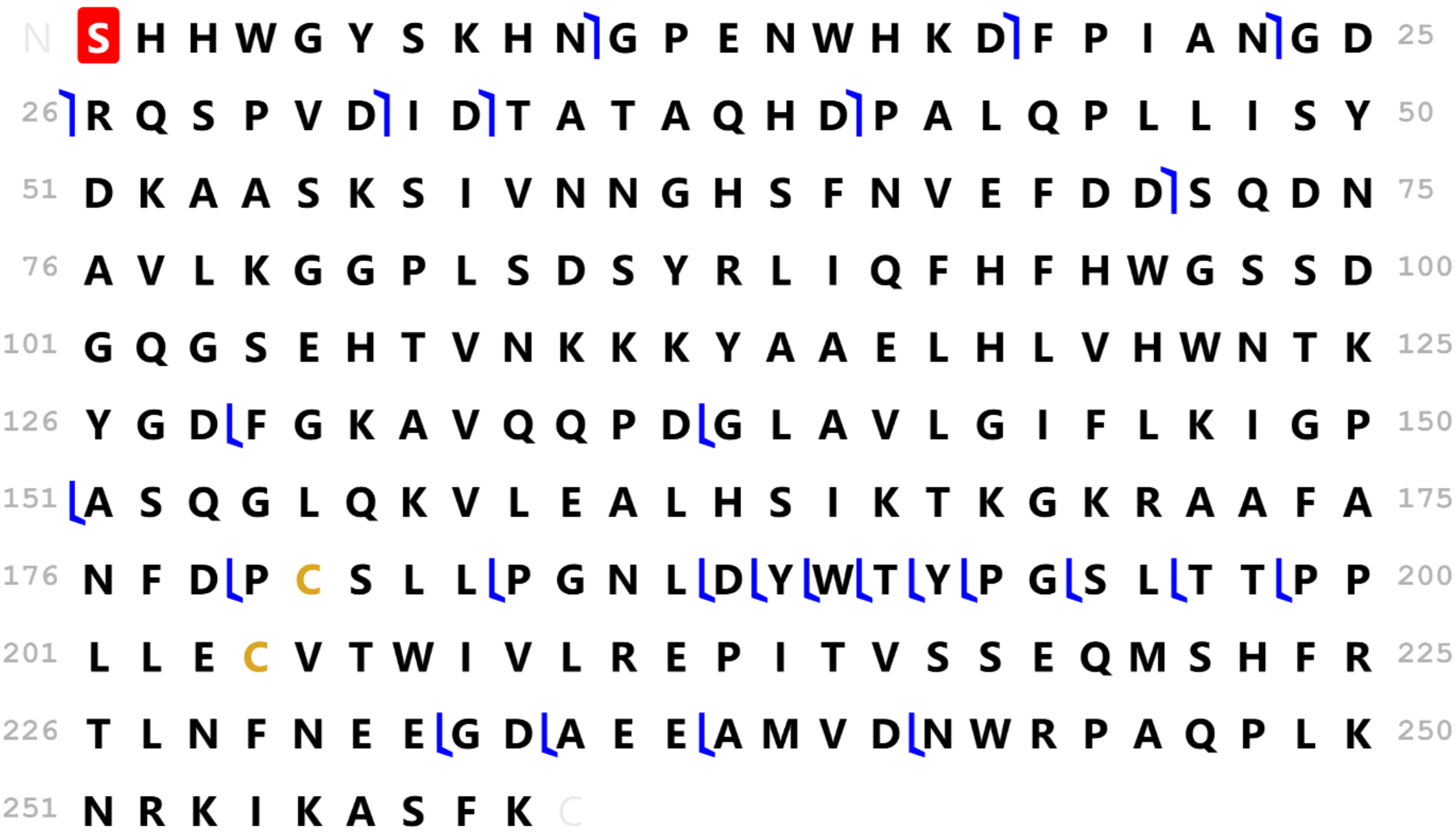

Figure S1. Sequence fragment map for CAH2 obtained following HCD MS^2 of m/z 2901.1 ±2.5.

Table S1. Fragment ions observed from CAH2 following HCD MS^2 of m/z 2901.1 ±2.5.

Fragment ion	Observed m/z	Charge state	Theoretical Mass	Observed Mass	Mass Difference (Da)	Mass Difference (ppm)
b10	1276.5658	1+	1275.5533	1275.5586	0.0052	4.09
b18	2239.9944	1+	2238.9732	2238.9871	0.0139	6.20
b25	2954.33	1+	2953.3069	2953.3227	0.0159	5.37
b31	1818.8392	2+	3635.6467	3635.6639	0.0172	4.72
b33	1932.9005	2+	3863.7577	3863.7865	0.0288	7.44
b40	2295.046	2+	4588.0717	4588.0774	0.0057	1.24
b40+Na	2306.0437	2+	4588.0717	4588.0898	0.0181	3.95
y18	2182.2813	1+	2181.2647	2181.2740	0.0093	4.27
y18	1091.6467	2+	2181.2647	2181.2789	0.0142	6.53
y22	1299.7338	2+	2597.4376	2597.4531	0.0155	5.96
y25	1464.2942	2+	2926.5599	2926.5739	0.0139	4.76
y27	1550.3159	2+	3098.6083	3098.6172	0.0089	2.88
y61	1774.6615	4+	7094.5996	7094.6170	0.0174	2.45
y63	1825.1837	4+	7296.6950	7296.7058	0.0108	1.48
y65	1875.2215	4+	7496.8111	7496.8567	0.0456	6.08
y67	2551.3102	3+	7650.8853	7650.9089	0.0235	3.08
y67	1913.7363	4+	7650.8853	7650.9162	0.0308	4.03
y68	1563.7965	5+	7813.9486	7813.9462	-0.0024	-0.31
y68	1954.4996	4+	7813.9486	7813.9692	0.0206	2.63
y69	1979.7639	4+	7914.9963	7915.0265	0.0302	3.82
y70	2026.2916	4+	8101.0756	8101.1374	0.0618	7.63
y72	2095.8187	4+	8379.1659	8379.2456	0.0797	9.51
y76	1753.0842	5+	8760.3671	8760.3848	0.0177	2.02
y76	2191.112	4+	8760.3671	8760.4190	0.0518	5.92
y81	2319.4248	4+	9273.6292	9273.6699	0.0407	4.39
y122	2261.3539	6+	13562.0867	13562.0799	-0.0068	-0.50
y131	2423.1133	6+	14532.57392	14532.6364	0.0625	4.30

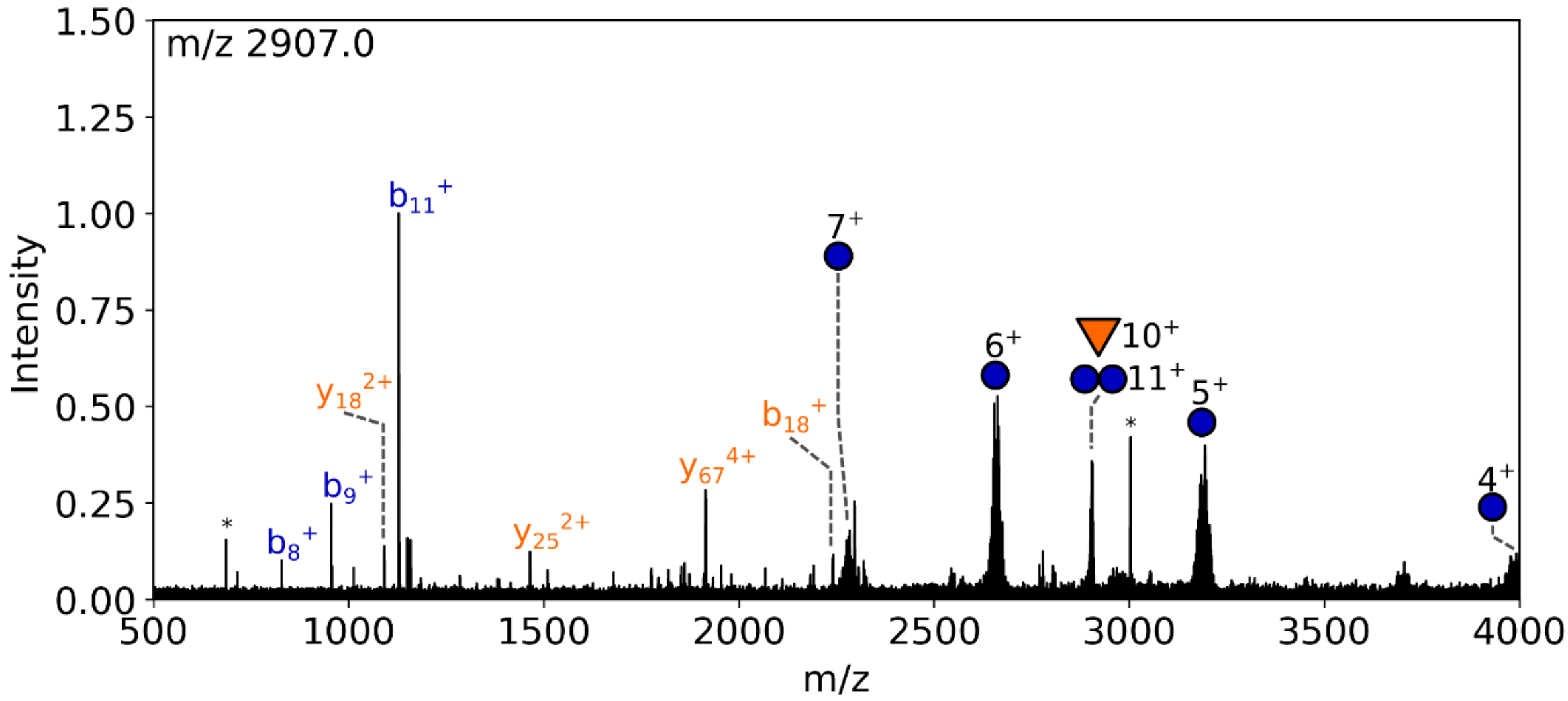

Figure S2. HCD MS^2 spectrum of m/z 2907 ±2.5 ions obtained from mouse spinal cord tissue section.

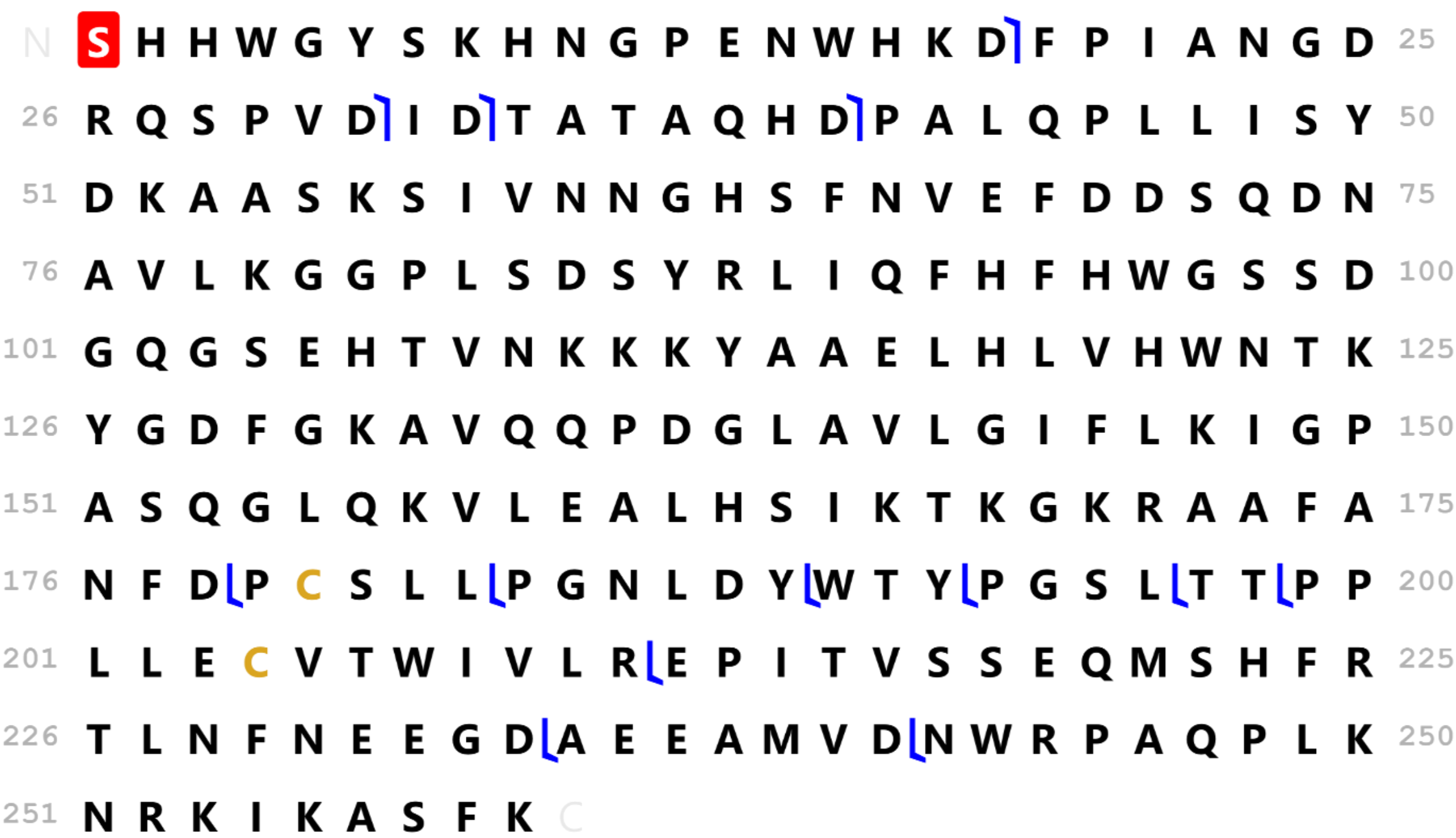

Figure S3. Sequence fragment map for CAH2 obtained following HCD MS^2 of m/z 2907 ±2.5.

Table S2. Fragment ions observed from CAH2 following HCD MS^2 of m/z 2907 ±2.5.

Fragment ion	Observed m/z	Charge state	Theoretical Mass	Observed Mass	Mass Difference (Da)	Mass Difference (ppm)
b18	2239.9860	1+	2238.9732	2238.9787	0.0055	2.46
b31	1818.8386	2+	3635.6467	3635.6627	0.0160	4.40
b33	1932.9007	2+	3863.7577	3863.7868	0.0291	7.54
b40	2295.0554	2+	4588.0717	4588.0963	0.0246	5.36
b40+Na	2306.0576	2+	4588.0717	4588.1176	0.0459	10.00
y18	1091.6427	2+	2181.2647	2181.2708	0.0061	2.80
y18	2182.2854	1+	2181.2647	2181.2781	0.0134	6.15
y25	1464.2949	2+	2926.5599	2926.5752	0.0153	5.22
y61	1774.6661	4+	7094.5996	7094.6353	0.0357	5.03
y67	1913.7308	4+	7650.8853	7650.8942	0.0088	1.15
y76	2191.1133	4+	8760.3671	8760.4243	0.0572	6.52
y81	2319.4106	4+	9273.6292	9273.6350	0.0058	0.62
y81+Na	2325.4260	4+	9273.6292	9273.6504	0.0212	2.29

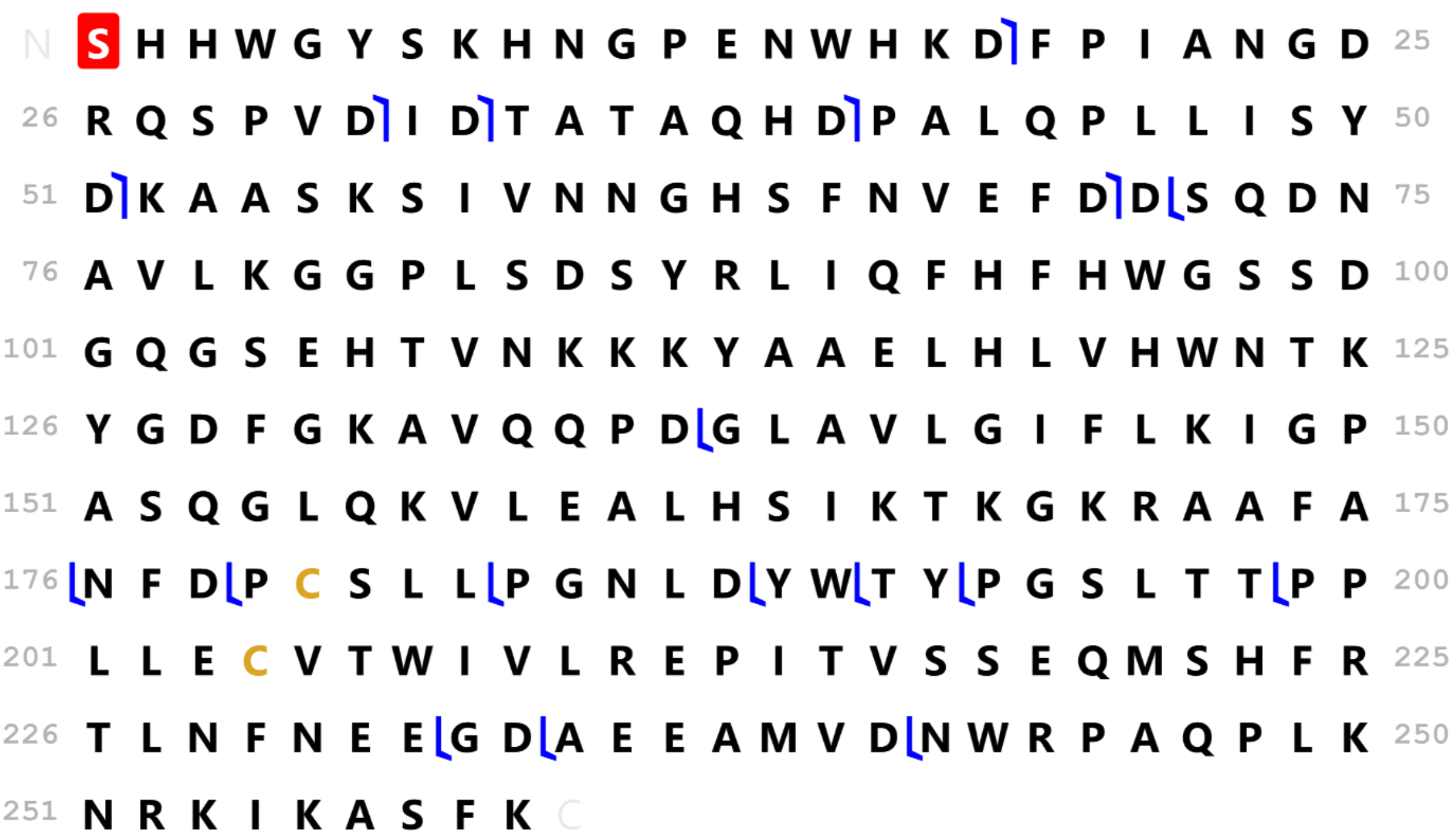

Figure S4. Sequence fragment map for CAH2 obtained following PTCR-HCD MS^3 of m/z 2901.1 ±2.5 → m/z 3224.

Table S3. Fragment ions observed from CAH2 following PTCR-HCD MS^3 of m/z 2901.1 ±2.5 → m/z 3224.

Fragment ion	Observed m/z	Charge state	Theoretical Mass	Observed Mass	Mass Difference (Da)	Mass Difference (ppm)
b18	2239.9937	1+	2238.9732	2238.9864	0.0132	5.91
b31	1818.8374	2+	3635.6467	3635.6602	0.0135	3.71
b31	3636.6733	1+	3635.6467	3635.6660	0.0193	5.32
b33	1932.8974	2+	3863.7577	3863.7802	0.0225	5.82
b40	2295.0454	2+	4588.0717	4588.0762	0.0045	0.97
b51	2900.3859	2+	5798.7317	5798.7572	0.0257	4.43
b70	3922.9009	2+	7843.7281	7843.7873	0.0592	7.55
y18	1091.6462	2+	2181.2647	2181.2778	0.0131	5.99
y25	1464.2925	2+	2926.5599	2926.5704	0.0105	3.58
y27	1550.3197	2+	3098.6083	3098.6248	0.0165	5.33
y61	1774.6619	2+	7094.5996	7094.61851	0.0188	2.66
y61	2365.8829	3+	7094.5996	7094.6267	0.0271	3.82
y67	1913.7352	4+	7650.8853	7650.9118	0.0265	3.46
y67	2551.3129	3+	7650.8853	7650.9170	0.0317	4.14
y69	1979.761	4+	7914.9963	7915.0150	0.0187	2.36
y69	2639.3573	3+	7914.9963	7915.0502	0.0539	6.80
y71	2067.0536	4+	8264.1390	8264.1853	0.0463	5.60
y71	2755.7364	3+	8264.1390	8264.1874	0.0484	5.86
y76	2191.1069	4+	8760.3671	8760.3986	0.0315	3.59
y81	3092.2238	3+	9273.6292	9273.6495	0.0203	2.19
y81	2319.423	4+	9273.6292	9273.6629	0.0336	3.63
y122	2713.4235	5+	13562.0867	13562.0813	-0.0054	-0.40

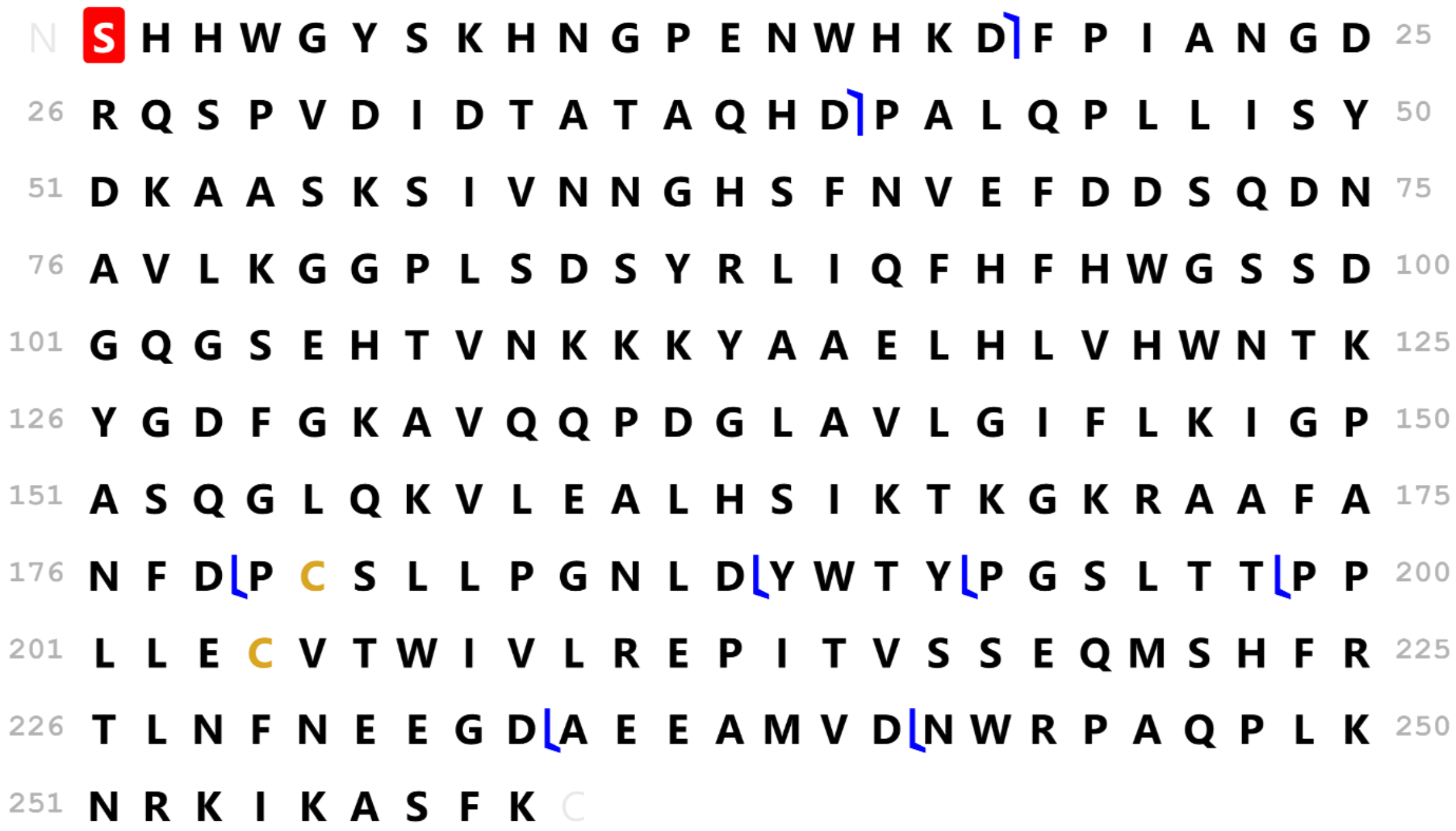

Figure S5. Sequence fragment map for CAH2 obtained following PTCR-HCD MS[3] of m/z 2907 ±2.5 → m/z 3228.

Table S4. Fragment ions observed from CAH2 following PTCR-HCD MS[3] of m/z 2907 ±2.5 → m/z 3228.

Fragment ion	Observed m/z	Charge state	Theoretical Mass	Observed Mass	Mass Difference (Da)	Mass Difference (ppm)
b18	2239.9811	1+	2238.9732	2238.9738	0.0006	0.28
b40	2295.0422	2+	4588.0717	4588.0699	-0.0018	-0.40
b40+Na	2306.0366	2+	4588.0717	4588.0761	0.0044	0.96
y18	1091.6383	1+	2181.2647	2181.2620	-0.0027	-1.22
y25	1464.2876	2+	2926.5599	2926.5605	0.0006	0.22
y61	1774.6528	4+	7094.5997	7094.5822	-0.0174	-2.46
y67	1913.7301	4+	7650.8853	7650.8912	0.0059	0.77
y67	2551.3142	3+	7650.8853	7650.9208	0.0355	4.63
y71	2755.7135	3+	8264.1390	8264.1187	-0.0203	-2.45
y81	2319.4142	4+	9273.6292	9273.6278	-0.0014	-0.15

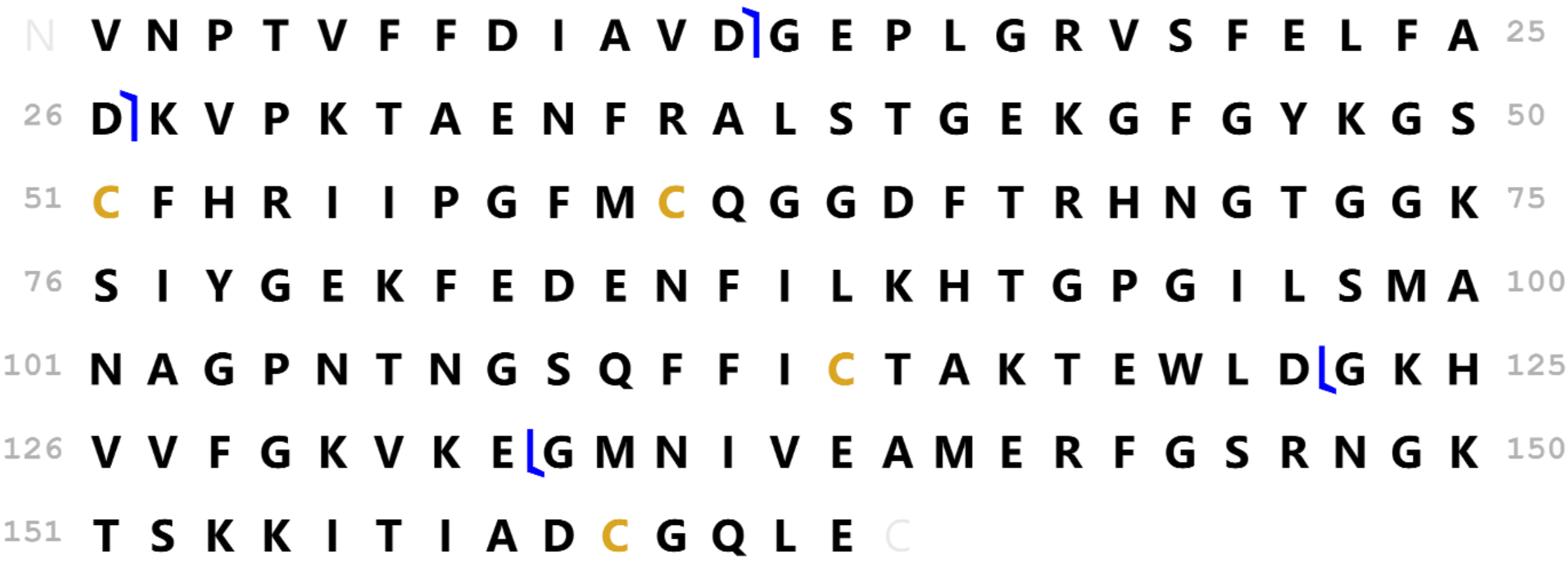

Figure S6. Sequence fragment map for glutathionylated PPIA (P62937) obtained following PTCR-HCD MS^3 of m/z 2594 ±10 → m/z 3032.

Table S5. Fragment ions observed from glutathionylated PPIA (P62937) obtained following PTCR-HCD MS^3 of m/z 2594 ±10 → m/z 3032.

Fragment ion	Observed m/z	Charge state	Theoretical Mass	Observed Mass	Mass Difference (Da)	Mass Difference (ppm)
b12	1318.6511	1+	1317.6605	1317.6439	-0.0166	-12.6
b26	2836.3897	1+	2835.4119	2835.3799	-0.0320	-11.3
y31	1692.8257	2+	3382.6795	3382.6340	-0.0455	-13.4
y42	2296.6702	2+	4591.3825	4591.3243	-0.0581	-12.7

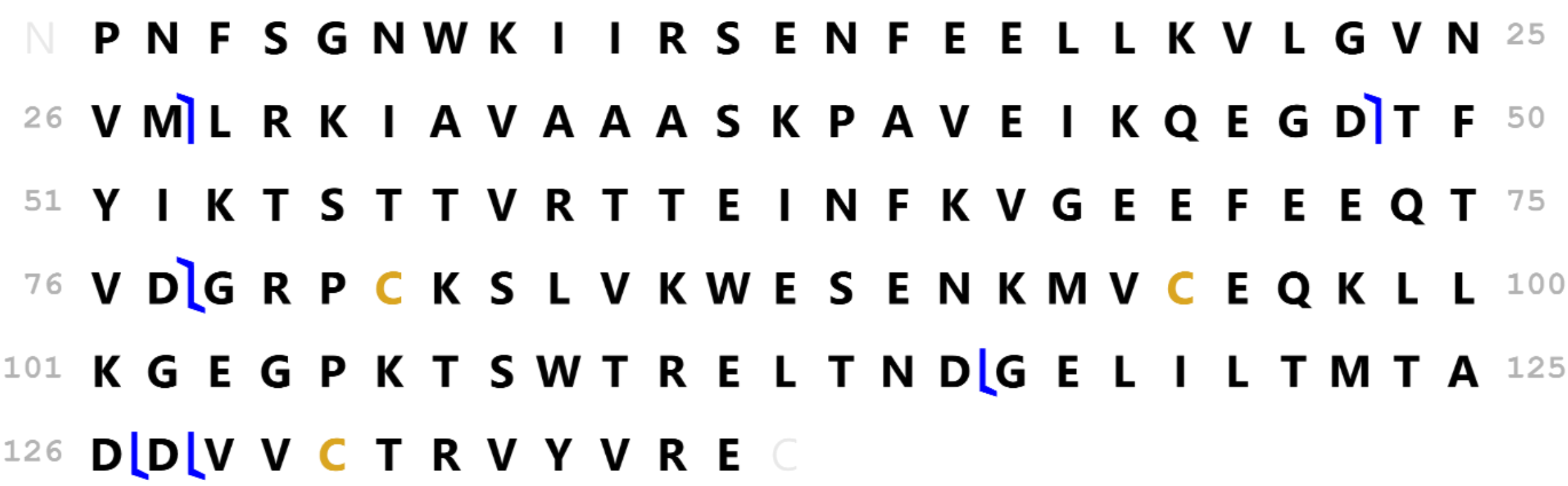

Figure S7. Sequence fragment map for RABP2 (P29373) obtained following PTCR-HCD MS^3 of m/z 2594 ±10 → m/z 3113.

Table S6. Fragment ions observed from RABP2 (P29373) obtained following PTCR-HCD MS^3 of m/z 2594 ±10 → m/z 3113.

Fragment ion	Observed m/z	Charge state	Theoretical mass	Observed Mass	Mass Difference (Da)	Mass Difference (ppm)
b48	2646.4067	2+	5289.8699	5289.8084	-0.0615	-11.6
b77	2896.1495	3+	8683.5315	8683.4210	-0.1105	-12.7
y10	1223.6400	1+	1222.64922	1222.6327	-0.0165	-13.5
y11	1338.6654	1+	1337.67616	1337.6582	-0.0180	-13.5
y21	2383.1721	1+	2382.1923	2382.1647	-0.0276	-11.6
y60	3436.2005	2+	6868.4482	6868.3733	-0.0749	-10.9

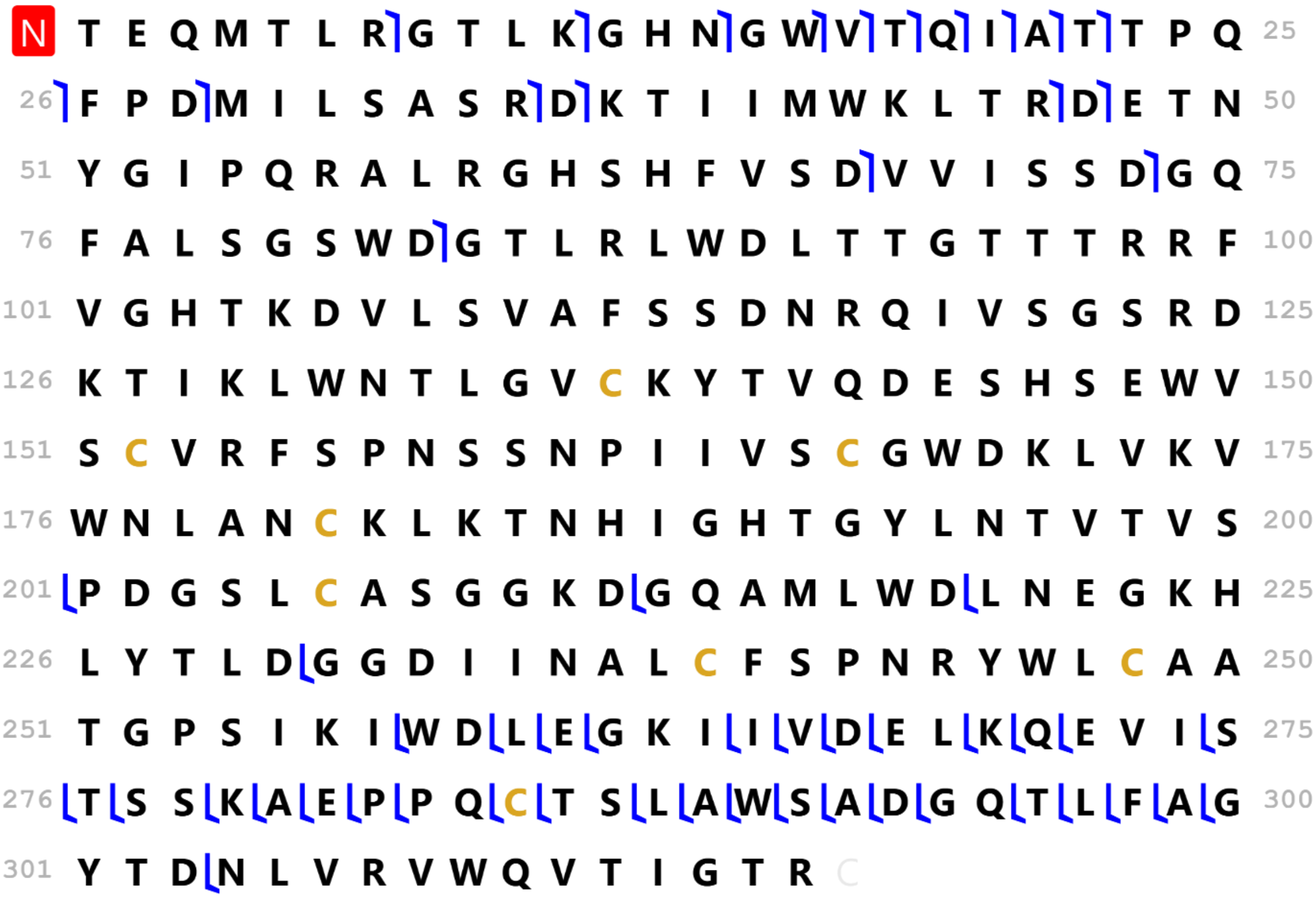

Figure S8. Sequence fragment map for RACK1 (P63245) obtained following HCD MS^2 of m/z 3181 ±5.

Table S7. Fragment ions observed from RACK1 (P63245) following HCD MS^2 of m/z 3181 ±5.

Fragment ion	Charge state	Theoretical mass	Observed mass	Mass Difference (Da)	Mass Difference (ppm)
b7	1+	901.4328	901.4315	-0.0012	-1.35
b11	1+	1300.6809	1300.6800	-0.0010	-0.73
b14	1+	1608.8042	1608.8014	-0.0029	-1.76
b16	2+	1851.9050	1851.9039	-0.0011	-0.57
b17	2+	1950.9734	1950.9708	-0.0026	-1.32
b18	2+	2052.0211	2052.0175	-0.0036	-1.76
b19	2+	2180.0797	2180.0755	-0.0042	-1.91
b20	2+	2293.1637	2293.159	-0.0045	-1.97
b21	2+	2364.2008	2364.1946	-0.0063	-2.65
b22	2+	2465.2485	2465.2430	-0.0055	-2.23
b25	1+	2791.4075	2791.4069	-0.0007	-0.24
b28	1+	3150.5556	3150.5549	-0.0007	-0.22
b36	2+	4023.9935	4023.9873	-0.0062	-1.54
b46	2+	5294.7518	5294.7366	-0.0152	-2.87
b47	2+, 3+	5409.7788	5409.7783	-0.0005	-0.09
b67	3+	7674.8827	7674.8777	-0.0049	-0.64
b73	3+, 4+	8275.1945	8275.1973	0.0028	0.34
b83	3+, 4+	9323.6559	9323.6396	-0.0163	-1.75
y13	1+, 2+	1540.8838	1540.8818	-0.0019	-1.26
y17	2+	1977.0432	1977.0400	-0.0032	-1.62
y19	2+	2195.1487	2195.1456	-0.0031	-1.43
y21	2+	2409.2804	2409.2743	-0.0061	-2.54
y23	1+, 2+	2594.3605	2594.3570	-0.0035	-1.34
y24	2+	2709.3874	2709.3820	-0.0054	-1.99
y25	2+	2780.4245	2780.4189	-0.0057	-2.03
y26	2+	2867.4566	2867.4523	-0.0042	-1.47
y27	2+	3053.5359	3053.5294	-0.0065	-2.12
y28	2+	3124.5730	3124.5675	-0.0055	-1.76
Y29	2+	3237.6570	3237.6522	-0.0048	-1.50
y31		3425.7368	3425.7307	-0.0061	-1.77
y32	2+	3528.7460	3528.7388	-0.0072	-2.03
y34	2+	3753.8573	3753.8523	-0.0050	-1.33
y35	2+, 3+	3850.9100	3850.9041	-0.0059	-1.54
y36	2+	3979.9526	3979.9401	-0.0125	-3.14
y37	2+	4050.9897	4050.9847	-0.0050	-1.23
y38	2+	4179.0847	4179.0765	-0.0082	-1.97
y40	2+	4353.1488	4353.1411	-0.0077	-1.76
y41	2+	4454.1964	4454.1930	-0.0034	-0.77
y42	2+	4541.2285	4541.2266	-0.0019	-0.42
y45	2+	4882.4235	4882.4219	-0.0017	-0.34

y46	2+	5010.4821	5010.4824	0.0002	0.048
y47	2+	5138.5771	5138.5794	0.0024	0.46
y49	2+, 3+	5380.7037	5380.7009	-0.0028	-0.53
y50	2+	5495.7307	5495.7429	0.0122	2.23
y51	2+, 3+	5594.7991	5594.7986	-0.0005	-0.09
y52	2+, 3+	5707.8831	5707.8807	-0.0024	-0.43
y55	3+	6006.0836	6006.0756	-0.0078	-1.29
y56	3+	6135.1262	6135.1166	-0.0097	-1.57
y57	2+, 3+	6248.2103	6248.2186	0.0083	1.33
y59	3+	6549.3165	6549.3184	0.0019	0.29
y86	3+, 4+	9410.7521	9410.7500	-0.0022	-0.23
y97	3+, 4+	10694.4031	10694.3991	-0.0040	-0.37
y104	3+, 4+, 5+	11495.7511	11495.7618	0.0108	0.94
y116	5+	12583.2115	12583.1874	-0.0241	-1.91

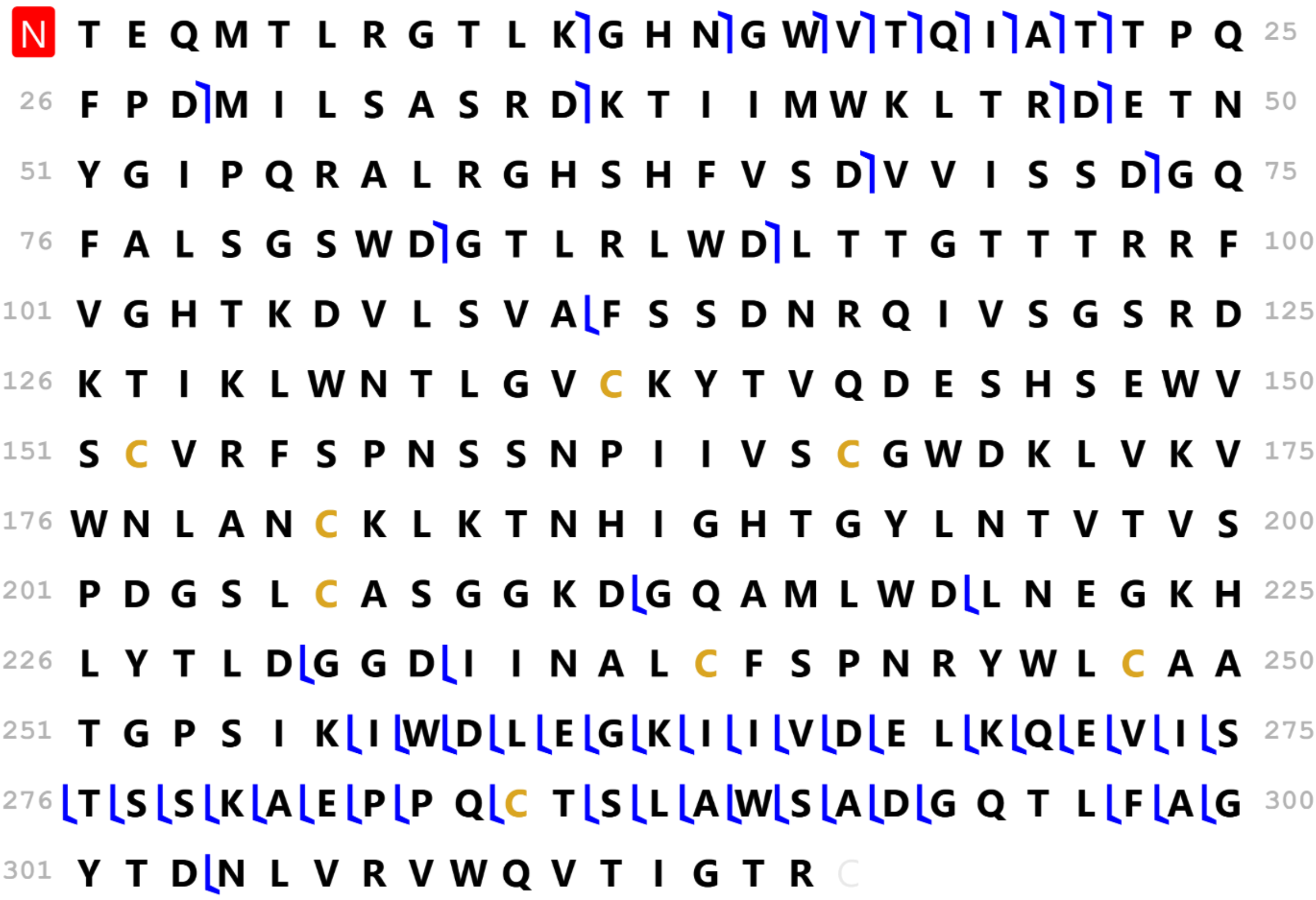

Figure S9. Sequence fragment map for RACK1 (P63245) obtained following PTCR-HCD MS^3 of m/z 3181 ±10 → m/z 3499.

Table S8. Fragment ions observed from RACK1 (P63245) following PTCR-HCD MS^3 of m/z 3181 ±10 → m/z 3499.

Fragment ion	Charge state	Theoretical mass	Observed mass	Mass Difference (Da)	Mass Difference (ppm)
b11	1+	1300.6809	1300.6779	-0.003	-2.33
b14	1+	1608.8042	1608.8004	-0.0038	-2.35
b16	2+	1851.9050	1851.9013	-0.0037	-2
b17	2+	1950.9734	1950.9670	-0.0064	-3.29
b18	2+	2052.0211	2052.0144	-0.0066	-3.23
b19	2+	2180.0797	2180.0735	-0.0061	-2.8
b20	2+	2293.1637	2293.1556	-0.0081	-3.52
b21	2+	2364.2008	2364.1927	-0.0082	-3.45
b22	2+	2465.2485	2465.2410	-0.0075	-3.04
b28	1+	3150.5556	3150.5542	-0.0015	-0.46
b36	2+	4023.9935	4023.9849	-0.0085	-2.12
b46		5294.7518	5294.7531	0.00131	0.248
b47	2+, 3+	5409.7788	5409.7709	-0.0079	-1.46
b67	3+	7674.8827	7674.8648	-0.0179	-2.33
b73	3+, 4+	8275.1945	8275.1825	-0.012	-1.45
b83	3+, 4+	9323.6559	9323.6311	-0.0248	-2.66
y13	1+, 2+	1540.8838	1540.8800	-0.0038	-2.48
y17		1977.0432	1977.0412	-0.002	-1.01
y18	2+	2048.0803	2048.074	-0.0063	-3.09
y19		2195.1487	2195.1415	-0.0072	-3.29
y23	1+, 2+	2594.3605	2594.3539	-0.0066	-2.53
y24		2709.3874	2709.3794	-0.0081	-2.97
y25	2+	2780.4245	2780.4164	-0.0082	-2.93
y26	2+	2867.4566	2867.4500	-0.0066	-2.3
y27	2+	3053.5359	3053.5279	-0.008	-2.61
y28	2+	3124.5730	3124.5638	-0.0092	-2.95
y29	2+	3237.6570	3237.6494	-0.0077	-2.37
y30	2+	3324.6891	3324.6818	-0.0073	-2.19
y32	2+	3528.7459	3528.7406	-0.0053	-1.51
y34	2+	3753.8573	3753.8491	-0.0082	-2.19
y35	2+	3850.9100	3850.9001	-0.01	-2.59
y36	2+	3979.9526	3979.9378	-0.0148	-3.72
y37	2+	4050.9897	4050.9803	-0.0094	-2.33
y38	2+	4179.0847	4179.0727	-0.012	-2.88
y39	2+	4266.1167	4266.1044	-0.0123	-2.88
y40	2+	4353.1488	4353.1434	-0.0053	-1.22
y41	2+	4454.1964	4454.1890	-0.0075	-1.68
y42	2+, 3+	4541.2285	4541.2204	-0.0081	-1.77
y43	2+, 3+	4654.3125	4654.2991	-0.0135	-2.89
y44	2+	4753.3809	4753.3726	-0.0083	-1.75

y45	2+	4882.4235	4882.4143	-0.0093	-1.9
y46	3+	5010.4821	5010.4747	-0.0074	-1.48
y47		5138.5771	5138.5814	0.00434	0.845
y49	3+	5380.7037	5380.6947	-0.009	-1.67
y50	3+	5495.7307	5495.7210	-0.0096	-1.76
y51	2+, 3+	5594.7991	5594.7826	-0.0165	-2.95
y52	3+	5707.8831	5707.8679	-0.0152	-2.67
y53	3+	5820.9672	5820.9505	-0.0167	-2.88
y54		5949.0622	5949.0415	-0.0207	-3.48
y55	3+	6006.0836	6006.0629	-0.0207	-3.45
y56	3+	6135.1262	6135.1094	-0.0168	-2.73
y57	2+, 3+	6248.2103	6248.2068	-0.0035	-0.56
y58	3+	6363.2372	6363.2263	-0.0109	-1.71
y59	3+	6549.3165	6549.3074	-0.0092	-1.4
y60	3+	6662.4006	6662.3909	-0.0096	-1.45
y83	3+	9181.6823	9181.6775	-0.0048	-0.52
y86	3+, 4+, 5+	9410.7521	9410.7443	-0.0079	-0.83
y97	4+, 5+	10694.4031	10694.3782	-0.025	-2.34
y104	4+, 5+	11495.7511	11495.7482	-0.0029	-0.25
